# Two Cases of Severe Hypertension in JAK2 Mutation-Positive Myeloproliferative Neoplasms

**DOI:** 10.1155/2020/8887423

**Published:** 2020-11-09

**Authors:** Raunak Rao, Spoorthy Kulkarni, Ian B. Wilkinson

**Affiliations:** Division of Experimental Medicine and Immunotherapeutics, University of Cambridge, Cambridge, UK

## Abstract

**Background:**

Myeloproliferative neoplasms are a heterogeneous group of disorders resulting from the abnormal proliferation of one or more terminal myeloid cells—established complications include thrombosis and haemorrhagic events; however, there is limited evidence to suggest an association with arterial hypertension. Herein, we report two independent cases of severe hypertension in JAK2 mutation-positive myeloproliferative neoplasms. *Case Presentations*. Case 1: a 39-year-old male was referred to our specialist hypertension unit with high blood pressure (BP) (200/120 mmHg), erythromelalgia, and headaches. We recorded elevated serum creatinine levels (146 *μ*M) and panmyelosis. Bone marrow biopsy confirmed JAK2-mutation-positive polycythaemia vera. Renal imaging revealed renal artery stenosis. Aspirin, long-acting nifedipine, interferon-alpha 2A, and renal artery angioplasty were employed in management. BP reached below target levels to an average of 119/88 mmHg. Renal parameters normalised gradually alongside BP. Case 2: a 45-year-old male presented with high BP (208/131 mmHg), acrocyanosis, (vasculitic) skin rashes, and nonhealing ulcers. Fundoscopy showed optic disc blurring in the left eye and full blood count revealed thrombocytosis. Bone marrow biopsy confirmed JAK2-mutation-positive essential thrombocytosis. No renal artery stenosis was found. Cardiac output was measured at 5 L/min using an inert gas rebreathing method, providing an estimated peripheral vascular resistance of 1840 dynes/s/cm^5^. BP was well-controlled (reaching 130/70 mmHg) with CCBs.

**Conclusions:**

These presentations highlight the utility of full blood count analysis in patients with severe hypertension. Hyperviscosity and constitutive JAK-STAT activation are amongst the proposed pathophysiology linking myeloproliferative neoplasms and hypertension. Further experimental and clinical research is necessary to identify and understand possible interactions between BP and myeloproliferative neoplasms.

## 1. Introduction

Myeloproliferative neoplasms (MPNs) are clonal disorders arising in the haematopoietic stem cell compartment. MPNs are mostly diagnosed in the age range of 50 to 60 years; however, presentations can occur in younger patients, especially with familial predisposition.

The most common MPN driver gene is Janus kinase 2 (JAK2), a cognate tyrosine kinase for both the erythropoietin and thrombopoietin receptors. JAK2-mutation-positive MPNs are associated with a higher rate of vascular complications. These include thrombotic events and occlusive vascular diseases. As of yet, there is limited evidence concerning a potential connection with arterial hypertension. However, several forms of vascular complications of MPNs have been described, including pulmonary hypertension [1], portal hypertension [2], and renovascular hypertension [3].

A retrospective analysis published in 2018 studying 1010 MPN patients in Malaysia demonstrated a significant association between JAK2 V617F mutation-positive MPNs and hypertension [4]. More recently, a study including 10 essential thrombocytosis patients with venous thrombosis found that all cases had concurrent arterial hypertension [5]. We report two cases of acute severe hypertension associated with JAK2 V617F mutation-positive MPNs. Earlier identification of MPNs in young, severely hypertensive patients may help in choosing the right treatment strategy leading to effective blood pressure (BP) control.

## 2. Case Presentations

Summaries of Cases 1 and 2 are displayed in Table 1.

### 2.1. Case 1 Polycythaemia Vera

A detailed account of Case 1 is displayed in Table 1.

Presentation: A 39-year-old male was referred to our specialist hypertension unit with a high BP (200/120 mmHg), erythromelalgia, and headaches.

**Figure 1:**
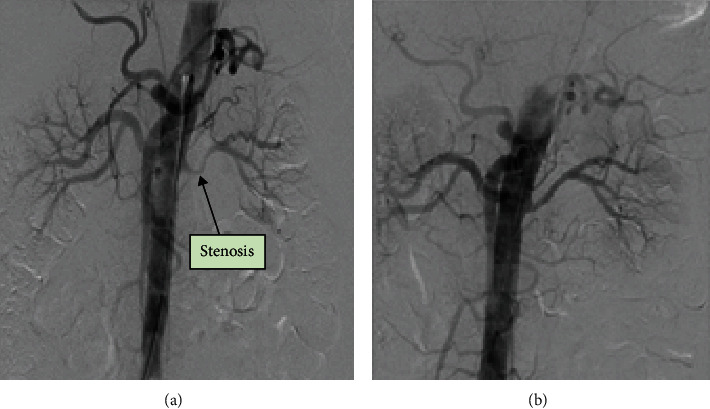
Case 1—(a) CT renal angiogram showing right renal artery before angioplasty. (b) CT renal angiogram showing right renal artery after angioplasty.

Investigations: We recorded elevated serum creatinine levels (146 *μ*M), panmyelosis with raised haematocrit (0.534 L/L), white blood cell (WBC) (15.8 × 10^9^/L) and platelet (PLT) (953 × 10^9^/L) counts. Bone marrow biopsy confirmed JAK2-mutation-positive polycythaemia vera. Renal imaging revealed renal artery stenosis (Figure 1(a)).

**Figure 2:**
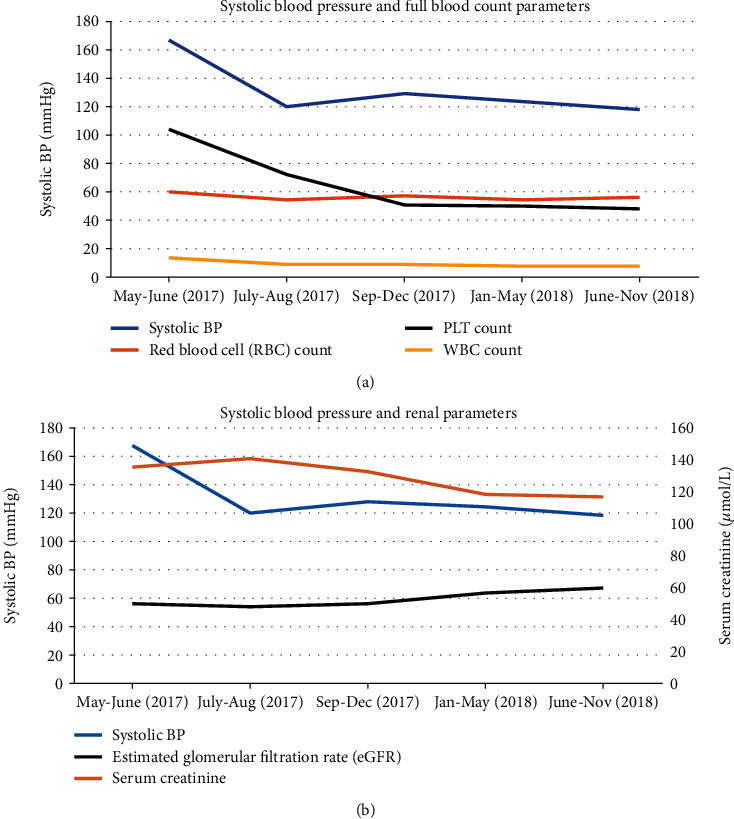
Case 1—(a) Changes in BP alongside FBC. (b) Changes in BP alongside renal parameters.

Outcomes and follow-up: Aspirin, nifedipine, interferon-alpha 2A (cytoreductive therapy—under haematology), and renal artery angioplasty (Figure 1(b)) were employed in management. BP reached below target levels to an average of 119/88 mmHg (Figure 2(a)). Renal parameters normalised gradually alongside BP (Figure 2(b)).

### 2.2. Case 2 Essential Thrombocytosis

**Figure 3:**
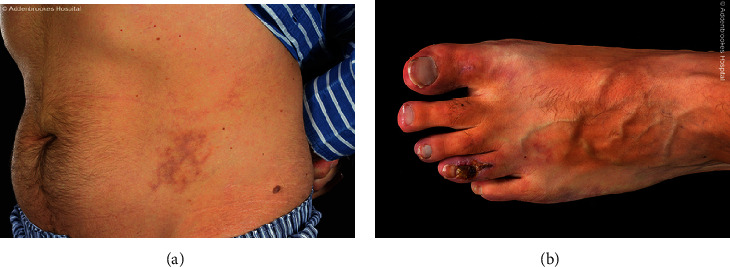
Case 2—(a) Vasculitic skin rashes. (b) Nonhealing ulcers.

Presentation: A 45-year-old male presented with high BP (208/131 mmHg), acrocyanosis, vasculitic skin rashes (Figure 3(a)), and nonhealing ulcers (Figure 3(b)).

Investigations: Fundoscopy showed optic disc blurring in the left eye and full blood count (FBC) revealed thrombocytosis (PLT -953 × 10^9^/L). Bone marrow biopsy confirmed JAK2-mutation-positive essential thrombocytosis.

Outcomes and follow-up—unlike our Case 1 patient, no renal artery stenosis was found. BP was well-controlled (reaching 130/70 mmHg) with nifedipine.

## 3. Discussion

Severely elevated BP (>180/120 mmHg) concomitant with new or progressive target organ dysfunction constitutes accelerated hypertension. Current practice overlooks FBC analysis when evaluating hypertension. Effective BP control was achieved in Case 1 and 2 with overlapping therapeutic interventions, suggesting a degree of similarity between the two pathophysiologies.

**Figure 4:**
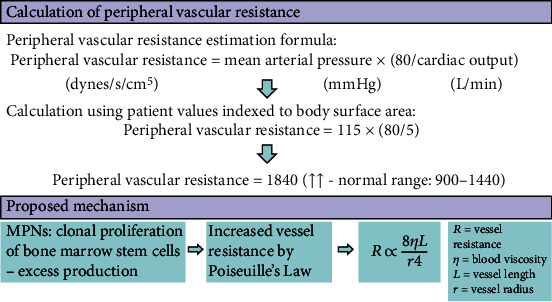
Case 2—Vessel resistance calculation and suggested mechanism for increase. Elevated peripheral vascular resistance with nonhealing ulcers could indicate vasoconstriction and microvascular rarefaction.

Both cases demonstrated good clinical responses to CCBs (over ACE inhibitor in Case 1), which cause direct vasodilatation through inhibition of calcium ion influx in vascular smooth muscle. This mechanism of action might be favourable in the context of hyperviscosity and elevated vascular resistance, as proposed in Figure 4. In Case 2, peripheral vascular resistance (PVR) was calculated from cardiac output and stroke volume measurements using an inert gas rebreathing method (Innocor) [6]. This provided an estimate of 1840 dynes/s/cm^5^, considerably exceeding the normal range of 900-1440 dynes/s/cm^5^, and that usually is seen in hypertension.

Raised PVR together with nonhealing ulceration could strongly suggest that vasoconstriction and microvascular rarefaction underly the hypotheses. A number of mechanisms may explain this in patients with essential thrombocytosis and polycythaemia vera. Increased haematocrit and platelet aggregation lead to whole blood hyperviscosity, which can add to both, the humoral and haemodynamic changes. Increased haematocrit increases the probability of adhesive wall collisions mediated by platelets. Platelets synthesise and discharge haemostatic mediators including thromboxane A2, PGF2 alpha, and serotonin, all of which are potent vasoconstrictors. These may lead to the activation of endothelial cells, resulting in increased expression of adhesion molecules and secretion of thrombogenic and angiogenic peptides from local inflammatory cells and procoagulant factors including von Willebrand factor (vWF). Endothelial injury/dysfunction has been postulated to be one of the mechanisms by which raised haematocrit predisposes to thrombotic complications [7]. Flow-mediated dilation (FMD), a marker for endothelial function, was found to be reduced in a small study in MPN patients, demonstrating the role of endothelium dysfunction [8]. Endothelial damage further leads to arterial insufficiency and capillary rarefaction—particularly in the microcirculation where endothelial dysfunction is greatest. This leads to a vicious cycle of endothelial damage, accounting for the chronic ulceration displayed by our patient.

The haemodynamic effects of ACE inhibitors and CCBs are known to differ. Whilst ACE inhibitors antagonise the renin-angiotensin system and reduce sympathetic output, CCBs mediate dilatation of large conduit and resistance arteries. These differences might explain the preferential responsiveness exhibited in both cases. However, the true relevance for MPN patients remains unclear, particularly as there is conflicting evidence to demonstrate a direct correlation between blood viscosity and pressure in normotensive and hypertensive subjects [9, 10].

Case 1 indicates an additional renovascular component to the patient's presentation. Several factors are likely to have contributed to the stenotic lesion including raised haematocrit, dysregulated coagulation, and abnormal vascular endothelial functions. These mechanisms have been discussed by Tamura et al. in a previous description of two patients with hypertension secondary to renal artery stenosis with JAK2 V617F-positive MPNs, both of whom were successfully managed with angioplasty [11]. The utility of angioplasty is considered highly dependent on the pathogenesis of renal artery stenosis. In patients with atherosclerotic stenosis, angioplasty demonstrates no significant advantage over medical management in the regulation of BP [12]. In comparison, MPN-associated renovascular hypertension responds well to this intervention. The greater extent of unresolved ischaemia in atherosclerotic pathophysiology is thought to account for this difference, meaning that MPN patients with coexistent renovascular atherosclerosis might receive limited benefit from renal angioplasty [13]. However, the fact that both patients responded to vasodilatory medications could imply that the stenosis is a bystander or indeed a by-product here rather than the main driver for acute rise in blood pressure.

Constitutive JAK-STAT activation has been suggested to increase the activation of platelets, and recruitment of platelets and leukocytes in conjunction with accelerated vascular cell hyperplasia [7]. Furthermore, the activated JAK-STAT pathway is known to contribute to local synthesis of Angiotensin II (Ang II), and in turn, the progression of Ang II-dependent hypertension [14]. Ang II-activated JAK2 prompts phosphorylation of Arhgef1 in vascular smooth muscle cells [15]; consequently, Arhgef1 stimulates the RhoA-Rho kinase axis, resulting in BP augmentation. In support of these findings, pharmacological inhibition of JAK2 has been shown to reduce hypertension in Ang-II infused animal models [16, 17]. JAK-STAT signalling has also been implicated in renal injury, with hyperglycaemia inducing the pathway via Ang II in glomerular mesangial cells [18]. Consistent with this observation, JAK2 inhibition in rat models prevented proteinuria and hypertension in streptozotocin-induced diabetic nephropathy [19], as well as ameliorating renal ischaemia-reperfusion injury [20].

Further study of the relationship between haemorheology, haemodynamics, and the role of endothelial dysfunction in MPNs is needed.

## 4. Conclusion

This report highlights the importance of simple FBC analysis in severe hypertension and the potential therapeutic role of CCBs. Increased platelet counts, hyperviscosity, rarefaction of vasculature, and resultant release of vasoconstrictive mediators are amongst the proposed pathophysiologies linking MPNs and hypertension. Associated vascular complications like arterial and venous thrombosis may add to the burden of vascular damage. Further experimental and clinical evidence is required to identify mechanisms of interaction between JAK2 function and BP.

## Figures and Tables

**Table 1:** Summary of case presentations.

	Case 1	Case 2
Initial BP (mmHg)	200/120	208/131
Presenting symptoms	Erythromelalgia, occipital headaches, Vertigo, one episode of dyspnoea + eye floaters + syncope (1-2 minutes)	Acrocyanosis, Vasculitic skin rashes, nonhealing toe ulceration
Past medical history	Spondylolisthesis	Migraines with aura
Family history	Father: Vasovagal syncope, myocardial infarction	Unremarkable
Social history	Ex-smoker	20 units of alcohol per week
Investigations and results		
Fundus examination	Papilloedema with nasal margin blurring	Arteriovenous nipping with nasal margin blurring
Urine dipstick	Unremarkable	Unremarkable
FBC	WBC—15.8 × 10^9^/L; RBC—6.20 × 10^9^/L; Hct -0.534 L/L PLT −953 × 10^9^/L; Neutrophil count −12.10 × 10^9^/L	PLT -1096 x10^9^/L
Liver function tests	Not indicated	Total bilirubin—38 *μ*mol/L; ALT—42 U/L; Gamma GT—94 U/L
Electrocardiogram	Sinus rhythm 70 bpm, left axis deviation, biphasic T waves in leads V5 and V6	Unremarkable
Echocardiogram	Moderate global left ventricular hypertrophy with 1.4 cm wall thickness	Mild aortic dilatation
Creatinine	146 *μ*M	88 *μ*M
Renin	107 mU/L	80 mU/L
Autoimmune screen	Negative	Negative
Vasculitic skin rash biopsy	N/A	Unremarkable
Contrast CT abdomen	N/A	Enlarged left adrenal gland, lower abdominal lymphadenopathy
Inert gas rebreathing studies (clinical research facility)	N/A	Peripheral vascular resistance -1840 dynes/s/cm^5^
Interventions	Renal artery angioplasty, aspirin 75 mg, intermittent venesection, interferon-alpha 2A	Aspirin 75 mg, long-acting nifedipine
Outcomes	Normalisation of BP and renal parameters, minor side effects from interferon therapy (decreased libido and reported thinning of hair), now managed with single dose of long-acting nifedipine	Normalisation of BP, no adverse outcomes reported, toe ulceration now healed

## Abbreviations

JAK2: Janus kinase 2
MPNs: Myeloproliferative neoplasms
BP: BP
WBC: White blood cell
PLT: Platelet
FBC: Full blood count
CT: Computerised tomography
RBC: Red blood cell
JAK-STAT: Janus kinase-signal transducer and activator of transcription
ACE: Angiotensin-converting enzyme
PVR: Peripheral vascular resistance
Ang II: Angiotensin II.
